# Endolymphatic Sac Drainage Surgery and Plasma Stress Hormone Vasopressin Levels in Meniere's Disease

**DOI:** 10.3389/fneur.2021.722217

**Published:** 2021-09-29

**Authors:** Tadashi Kitahara, Tadao Okayasu, Taeko Ito, Hiroto Fujita, Keita Ueda

**Affiliations:** Department of Otolaryngology-Head and Neck Surgery, Nara Medical University, Nara, Japan

**Keywords:** Meniere's disease, endolymphatic hydrops, endolymphatic sac drainage, steroids, stress hormone, vasopressin

## Abstract

Meniere's disease is a common inner ear disorder accompanied by vertigo attacks and fluctuating hearing loss that some believe is due to a stressful lifestyle. To elucidate the scientific relationship in neuro-endocrinology between Meniere's disease and stress, we examined the surgical results of endolymphatic sac drainage surgery and changes in stress-induced plasma arginine-vasopressin levels. We enrolled 100 intractable Meniere's patients and examined surgical results and plasma vasopressin levels. Fifty-four chronic otitis media patients who underwent tympano-mastoidectomy formed a control group. We assessed surgical results during a 2-year follow-up period, including vertigo and hearing loss. We examined plasma vasopressin levels just before surgery and 1 week, 1 year, and 2 years after surgery. In patients with intractable Meniere's disease, plasma vasopressin levels were significantly reduced 1 week after surgery compared to the decrease observed in chronic otitis media patients after tympano-mastoidectomy. In intractable Meniere's disease, long-lasting low plasma vasopressin levels after surgery were associated with significantly good surgical results. In recurrent Meniere's disease, a gradual plasma vasopressin level elevation was observed after surgery, followed by recurrent vertigo attacks and sensorineural hearing loss. It is suggested that long-lasting high levels of plasma vasopressin could have adverse effects on inner ear water metabolism and the subsequent Meniere's disease symptoms. Effective treatments for Meniere's disease might be best based on the maintenance of low plasma vasopressin levels.

## Introduction

Over time, animals with receptors that detect only environmental vibrations have evolved into higher animals that have increased mobility and can respond to sound and vibration. Based on the need for better auditory and equilibrium perception, the semicircular canals and lagena, and then the cochlea, developed from the primitive otolith. Fish are already equipped with semicircular canal-like structures, but not with a cochlea. In mammals, the utricular and oval macula, ampullar crista, and inner and outer hair cells in the organ of Corti are specialized pressure receptors. These receptors in the inner ear have evolved so that different types of pressures, such as linear and angular acceleration and sound, can be detected (1). Throughout the process of biological evolution, these receptors inside the inner ear have been housed in an aquatic environment, although additional structures, such as the middle ear and Eustachian tube, are formed around the inner ear to work these receptors more effectively according to environmental changes.

Even in human inner ear receptors that have evolved to effectively respond to the environment, disruption of the water metabolism of the inner ear can sometimes cause dysfunction. This kind of dysfunction sometimes leads to repeated episodes of vertigo and fluctuating hearing loss and tinnitus, that is, Meniere's disease. This syndromic triad has been described since the Hippocratic era, and audio-vestibular diseases that result from excessive water in the whole body have also been described throughout China's 2000-year history of Kampo medicine. Surprisingly, relationships between the inner ear function and local water metabolism were clarified much later in the 17th−18th century, when Cotuguno and Scarpa found the membranous labyrinth filled with endolymphatic fluids. Thereafter, in the 20th century, Yamakawa in Osaka (2) and Hallpike and Cairns in London (3) revealed inner ear endolymphatic hydrops as the oto-pathology in Meniere's disease.

**Figure 1:**
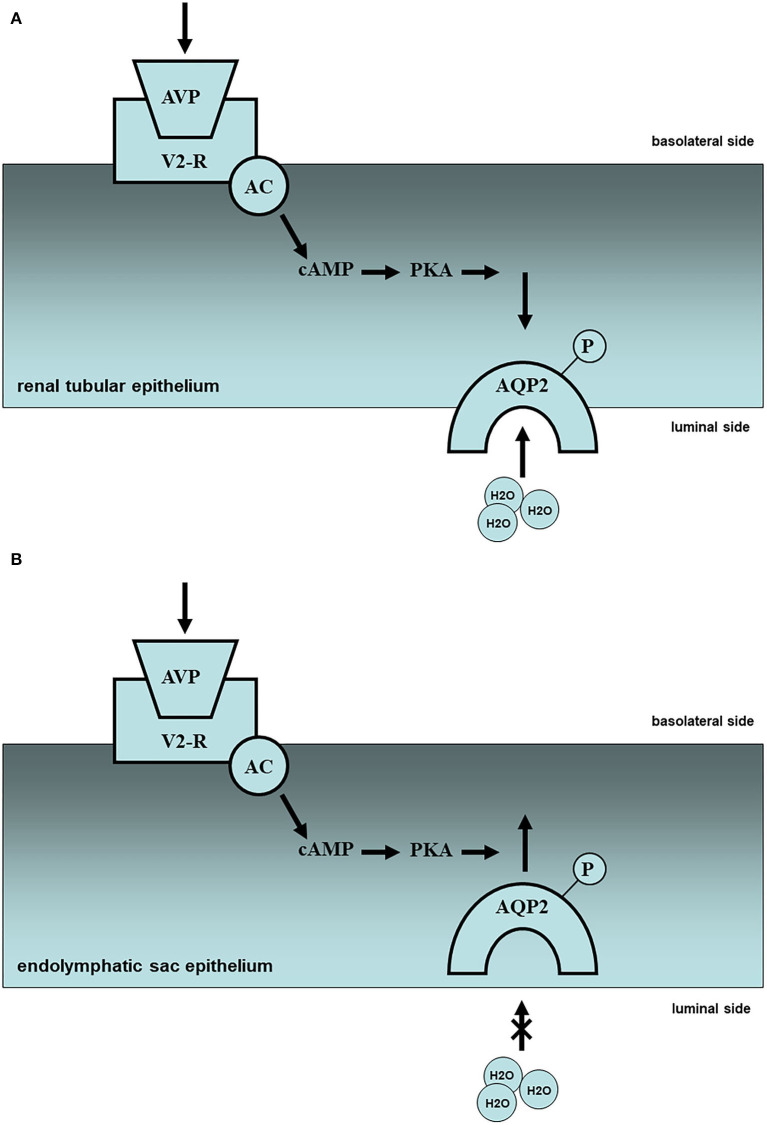
Molecular mechanism of water metabolism in the kidney and endolymphatic sac (partial hypothesis). **(A)** In the renal tissues, plasma vasopressin (AVP) elevation and subsequent V2 receptor (V2R)-cyclic AMP (cAMP)-protein kinase A (PKA) activation in the renal tubule might lead the intracellular translocation of aquaporin-2 (AQP2) from basolateral side to luminal side, resulting in the reabsorption of urine, i.e., oliguria. Upward side: basolateral side; downward side: luminal side. **(B)** In the endolymphatic sac tissues, plasma vasopressin (AVP) elevation, and subsequent V2 receptor (V2R)-cyclic AMP (cAMP)-protein kinase A (PKA) activation in the endolymphatic sac might lead the intracellular translocation of aquaporin-2 (AQP2) from luminal side to basolateral side with endosomal trapping, resulting in the pathogenesis of inner ear endolymphatic hydrops, i.e., Meniere's disease. Upward side: basolateral side; downward side: luminal side.

Fluid homeostasis in the inner ear is dependent upon the production, transport, and absorption of water, similar to the kidneys. Thus, localized arginine-vasopressin (AVP), AVP-related molecules such as vasopressin receptors, and water channels or aquaporins (AQPs) are present in the inner ear (4, 5). AVP is produced in the hypothalamus-pituitary axis and taken to the target organs by blood flow. AVP acts on vasopressin receptor V1a (V1a-R) in the vascular epithelium and activates phospholipase C, resulting in the regulation of calcium ions and the vaso-diameter. AVP acts on vasopressin receptor V2 (V2-R) in the basolateral side of the vascular epithelium and activates adenylate cyclase (AC), resulting in the regulation of water absorption through cyclic AMP (cAMP) elevation with protein kinase A (PKA) activation (Figure 1).

V1a-R is localized not only in the stria vascularis but also in the endolymphatic sac. However, the role of V1a in the inner ear remains unclear. V2-R plays a pivotal role in water metabolism in the inner ear, as well as in the kidney through the AVP-V2R-AC-cAMP system mentioned above. In the renal tissues plasma AVP (pAVP) elevation and subsequent V2R-cAMP-PKA activation in the renal tubule might lead to the intracellular translocation of AQP2 from the basolateral side to the luminal side of the tubule, resulting in the reabsorption of urine (Figure 1A). In the endolymphatic sac tissues, pAVP elevation and subsequent V2R-cAMP-PKA activation in the endolymphatic sac might lead to intracellular translocation of AQP2 from the luminal side to the basolateral side of the endolymphatic sac with endosomal trapping, resulting in the pathogenesis of inner ear endolymphatic hydrops (Figure 1B). In injuries with massive bleeding, the water resorption system may be promoted in the renal tubules to keep adequate systemic blood flow and inhibited in the inner ear to prevent endolymphatic collapse. AVP application to the inner ear tissues could activate V2-R and AC with cAMP elevation and AQP2 translocation (6, 7), resulting in the inner ear endolymphatic hydrops (8, 9). Despite no histopathological findings in the inner ear using V2-R knock-out mice, there is no doubt that V2-R could play a pivotal role in water permeability in the inner ear, including the endolymphatic sac.

Temporal bone studies conducted in 1938 revealed that the pathological feature of Meniere's disease is endolymphatic hydrops (2, 3). Meniere's disease is a common disorder with an incidence of 15–50 per 100,000 persons. It is suggested that it occurs particularly in civilized people living a stressful lifestyle, one author has termed “Menierization is civilization” (10). AVP is one of the stress-related hormones, and therefore, it is associated with both stress and the development of inner ear endolymphatic hydrops. High levels of pAVP in patients with Meniere's disease have been reported since the 1990s (6, 11, 12), although this topic has been controversial with contrary findings (13).

Given the role of AVP in the renal tubules, it is logical that continuously high levels of pAVP could result in adverse effects on the inner ear fluid homeostasis, resulting in cochlear and vestibular dysfunction. However, the reports are limited to the notion that AVP, as one of the stress-related hormones secreted from the hypothalamus-pituitary axis, is associated only with physical stress. Therefore, the suggestion that high levels of plasma stress hormone AVP in patients with Meniere's disease could cause vertigo attacks or stressful vertigo attacks has remained controversial. This study aimed to investigate the scientific relationship in neuro-endocrinology between Meniere's disease and stress and whether elevated pAVP levels in Meniere's disease are a cause or a consequence of vertigo attacks.

## Materials and Methods

The present study was approved by the Ethics Committee of Osaka University Hospital (certificate number: 0421) and registered with ClinicalTrials.gov of the U.S. Food and Drug Administration (certificate number: NCT00500474).

### Patients and Controls

One hundred patients (male/female = 42/58; age = 51.5 ± 15.1 years) with intractable Meniere's disease—diagnosed according to the 1995 American Academy of Otolaryngology–Head and Neck Surgery (AAO-HNS) criteria (14)—were enrolled in the present study between 1997 and 2006. These cases received endolymphatic sac drainage surgery with intra-endolymphatic sac local steroids (endolymphatic sac drainage surgery group: R44/L56), as previously described (15, 16). Another 54 cases with chronic otitis media without any direct inner ear damage acted as a control group (male/female = 21/33; age = 54.2 ± 18.8 years) and received tympano-mastoidectomy (tympano-mastoidectomy group: R24/L30). There were no significant differences in patient background between the two groups.

The 100 patients with intractable Meniere's disease (unilateral: 77; bilateral: 23) received 100 endolymphatic sac drainage surgeries, and their surgical results regarding vertigo and hearing were evaluated according to the 1995 AAO-HNS criteria (14). These patients were divided into a complete group (*n* = 48: zero vertigo and more than 10 dB improvement in hearing) and a non-complete group (*n* = 52: other). Short-term (15) and long-term results of this surgery (16) were described previously. In detail, the non-complete group was composed of those with zero vertigo/no change in hearing (*n* = 40), those with recurrent vertigo/no change in hearing (*n* = 5), and those with recurrent vertigo/more than 10 dB deterioration in hearing (*n* = 7).

### Laboratory Examinations

Before collecting blood samples, all patients provided informed consent. Blood samples were collected from both groups at 8:00 a.m.−10:00 a.m. on the day of surgery and at 1 week, 1 year, and 2 years after surgery. Blood for the pAVP assay was transferred to an ethylenediaminetetracetic acid tube and centrifuged at 4°C, and the separated plasma was stored at −80°C. The pAVP level was determined by radioimmunoassay (arginine vasopressin radioimmunoassay kit: Mitsubishi, Tokyo, Japan) (6, 12).

### Statistical Analysis

Statistical differences in the patient background (sex and age) between patients with Meniere's disease and controls were examined using a Mann–Whitney *U*-test. Statistical differences between the two groups in terms of outcomes were determined using an unpaired *t*-test, one-way analysis of variance (ANOVA), or two-way repeated ANOVA. All reported *p*-values were two-sided and those under 0.05 were considered significant. All statistical analyses were performed using SPSS version 20.0 (SPSS Inc., Chicago, IL, USA).

## Results

**Figure 2:**
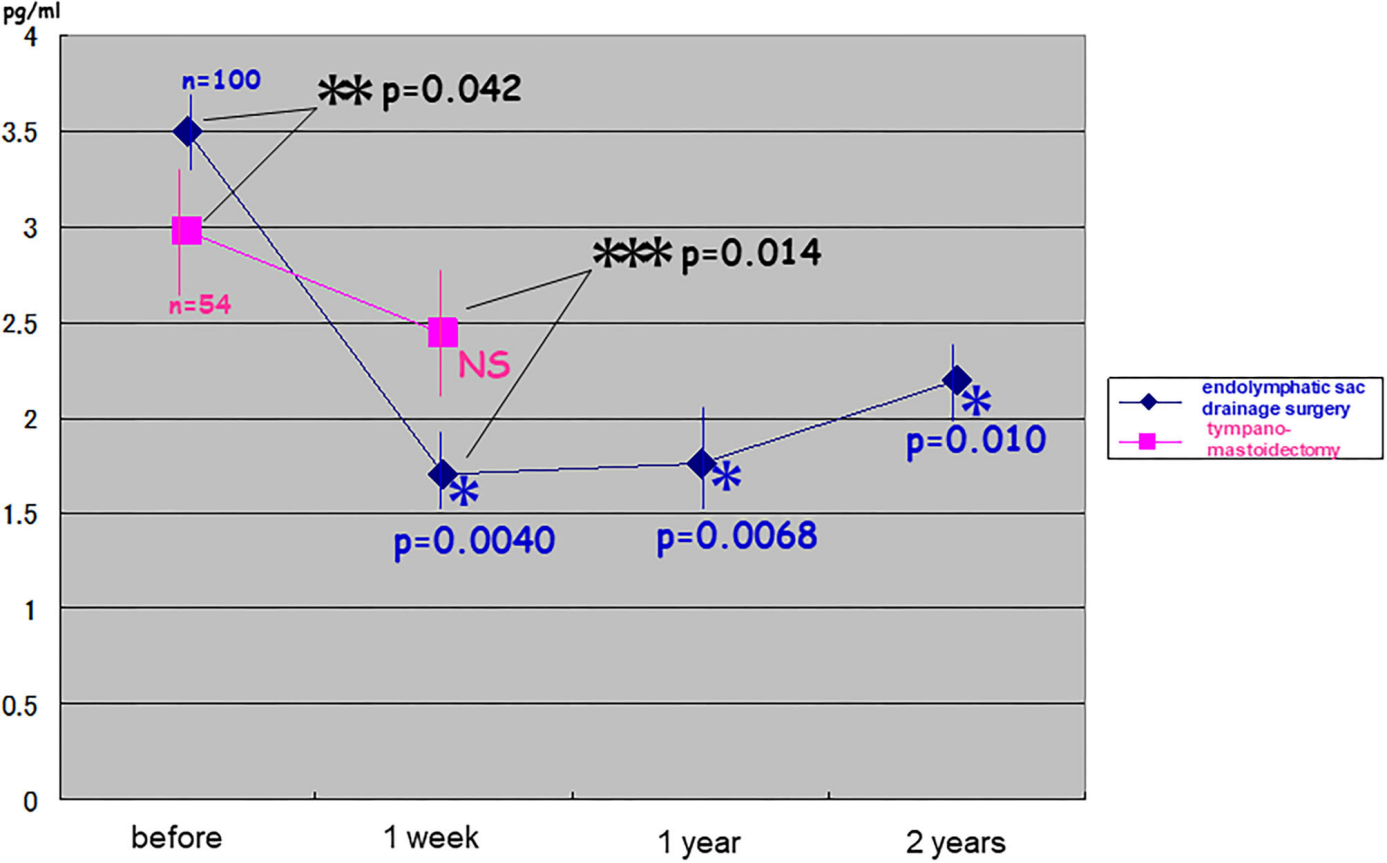
Changes in plasma stress hormone vasopressin after endolymphatic sac drainage surgery vs. tympano-mastoidectomy. Plasma vasopressin (pAVP) in intractable Meniere's disease was significantly higher than that in chronic otitis media before surgery (*t*-test: *p* = 0.042 < 0.05). It was reduced immediately after endolymphatic sac drainage surgery (one-way ANOVA: *p* = 0.0040 < 0.005) and as was much greater than that in chronic otitis media after tympano-mastoidectomy (two-way repeated ANOVA: *p* = 0.014 < 0.05). The significantly lower levels of pAVP lasted at least for 2 years after endolymphatic sac drainage surgery (one-way ANOVA: *p* = 0.0068 < 0.01; *p* = 0.010 < 0.05).

Before the surgery, pAVP levels were significantly higher in patients with intractable Meniere's disease than in patients with chronic otitis media (*t*-test: *p* = 0.042). pAVP levels were significantly reduced immediately after endolymphatic sac drainage surgery (one-way ANOVA: *p* = 0.0040), and this reduction was much greater than that in patients with chronic otitis media after tympano-mastoidectomy (two-way repeated ANOVA: *p* = 0.014). Significantly lower pAVP levels remained for at least 2 years after endolymphatic sac drainage surgery [one-way ANOVA: *p* = 0.0068 (compared to immediately after surgery) and *p* = 0.010 (compared to patients with chronic otitis media after tympano-mastoidectomy)] (Figure 2).

**Figure 3:**
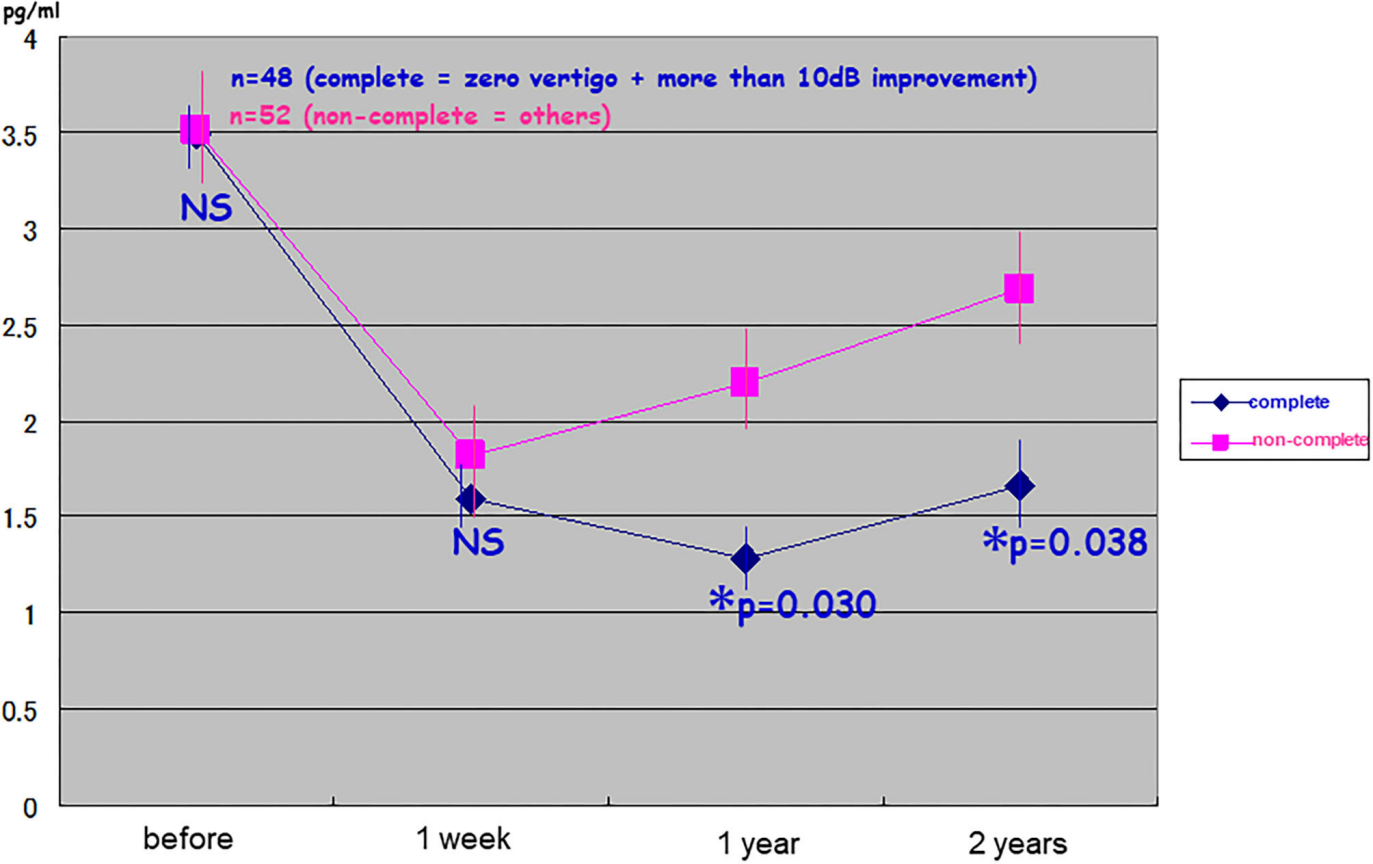
Relationship between long-lasting suppression of plasma stress hormone vasopressin and good results in endolymphatic sac drainage surgery. One hundred patients with intractable Meniere's disease received endolymphatic sac drainage surgery were divided into the complete group (*n* = 48: zero vertigo and more than 10 dB improvement) and non-complete group (*n* = 52: others). The cases with good surgical results in vertigo and hearing at the 2nd post-operative year showed significantly lower levels of plasma vasopressin (pAVP) at least for 2 years predictively. ^*^Significant (*p* < 0.05); NS, not significant.

Patients with good surgical results in terms of vertigo and hearing at the second post-operative year showed significantly low levels of pAVP at least for 2 years post-operatively (the complete pAVP: 1.67 ± 0.26; the non-complete pAVP: 2.71 ± 0.30; Figure 3). In detail, the non-complete group was divided into three, resulting in the averaged pAVP: 2.55 ± 0.48 of zero vertigo/no change in hearing, the averaged pAVP: 4.50 ± 1.43 of recurrent vertigo/no change in hearing, and the averaged pAVP: 4.78 ± 1.88 of recurrent vertigo/more than 10 dB deterioration in hearing (no statistical significance among three).

**Figure 4:**
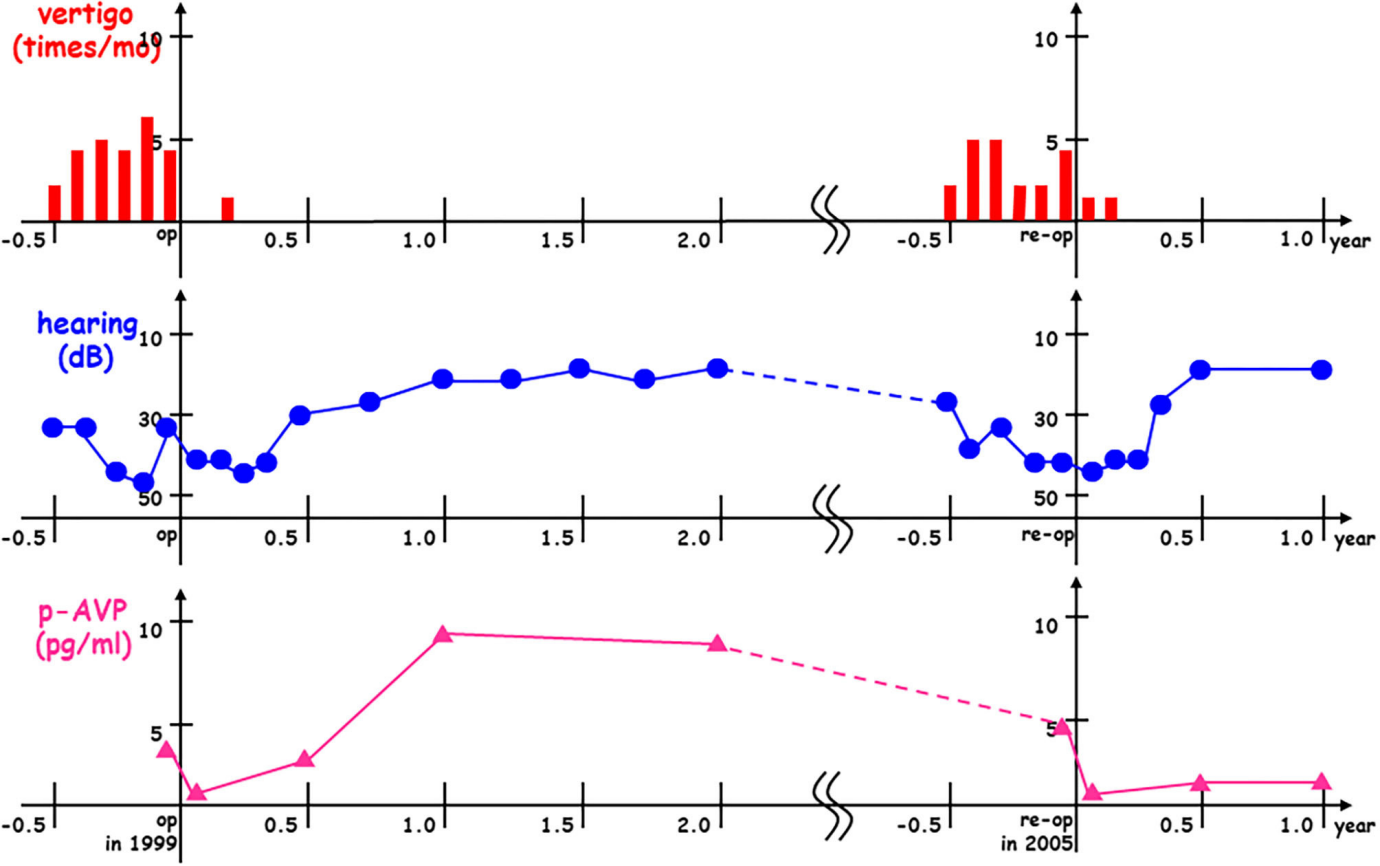
Plasma stress hormone vasopressin re-elevation and subsequent Meniere's recurrence after endolymphatic sac drainage surgery. In this case with interactable Meniere's disease, plasma vasopressin (pAVP) was reduced immediately after the 1st endolymphatic sac drainage surgery in 1999 (op in 1999) and then vertigo (times/mo) and hearing (dB) were gradually improved. After a while, pAVP re-elevation was observed at the 1st post-operative year and then followed by recurrent vertigo attack and sensorineural hearing loss at the 6th post-operative year. Finally, this case with recurrent Meniere's disease received the 2nd endolymphatic sac drainage surgery in 2005 (re-op in 2005) to get pAVP dropped down with gradual improvement vertigo and hearing again.

In one of the typical patients with intractable Meniere's disease, pAVP levels were reduced immediately after the first endolymphatic sac drainage surgery in 1999 and then vertigo and hearing gradually improved. After a while, pAVP re-elevation was observed during the first post-operative year and then followed by a recurrent vertigo attack and sensorineural hearing loss during the sixth post-operative year. Finally, this patient with recurrent Meniere's disease underwent a second endolymphatic sac drainage procedure in 2005, resulting in reduced pAVP levels and gradual improvement in vertigo and hearing again (Figure 4).

## Discussion

### Plasma Vasopressin Elevation: Cause or Consequence of Meniere's Attacks?

Takeda et al. (11) first paid attention to high levels of pAVP in patients with Meniere's disease in the 1990s, although this topic has been controversial with contrary findings (13). In detail, patients with a vestibular type of Meniere's disease showed low levels of pAVP because of a combination with non-endolymphatic hydrops, and those with long-term profound sensorineural hearing loss demonstrated high levels of pAVP because they were candidates for delayed endolymphatic hydrops, suggesting relationships between continuous high levels of pAVP and endolymphatic hydrops genesis.

Takeda et al. also demonstrated that guinea pigs with chronic intraperitoneal application of AVP presented with cochlear endolymphatic hydrops with auditory brainstem response deterioration (8, 9). Moreover, Maekawa et al. (7) revealed that human endolymphatic sac tissues cultured with AVP application resulted in cAMP activation and AQP2 translocation from the luminal side to the basolateral side of the endolymphatic sac to reduce endolymphatic absorption. These findings suggest that elevated AVP levels possibly cause endolymphatic hydrops and subsequent inner ear dysfunction.

In the present study, in intractable cases of Meniere's disease, long-lasting low pAVP levels after surgery were associated with significantly better surgical results. In recurrent cases with Meniere's disease, as seen in Figure 4, gradual pAVP elevation was initially observed after surgery, and this was followed by a recurrent vertigo attack and sensorineural hearing loss. Furthermore, in our recent inner ear magnetic resonance imaging (MRI) study, endolymphatic volume decreased after the surgery, resulting in significantly better surgical results (17). These findings suggest that long-lasting high levels of pAVP could have adverse effects on the inner ear water metabolism and possibly cause subsequent Meniere's symptoms.

On the contrary, in clinical studies, patients with a syndrome of inappropriate secretion of antidiuretic hormone and high levels of pAVP do not always have endolymphatic hydrops or Meniere's disease symptoms. There are some cases with low pAVP levels who are diagnosed with Meniere's disease. In basic studies there are no reports of endolymphatic hydrops or collapses in the inner ear of AVP- or V2R-deficient mice. These findings suggest that inner ear fluid homeostasis may work under complicated intracellular signal transduction cascades, including AVP-V2R.

### Stress Hormone Management: A Possible Treatment Strategy for Meniere's Disease

Persistent high levels of pAVP could have adverse effects on the endolymph as a cause of endolymphatic hydrops and the onset of Meniere's symptoms. Consequently, efforts should be made to maintain low levels of pAVP to prevent disease development.

Takahashi et al. (18) reported the results of their questionnaire study, demonstrating that patients with Meniere's disease have characteristics associated with a type A personality, including operating at a more urgent pace, demonstrating higher levels of impatience, having a more competitive nature, getting upset easily, and associating self-worth with achievement. Although this study did not measure pAVP, it suggests that psychological counseling aimed at promoting self-awareness may result in some level of symptomatic control. Naganuma et al. (19) suggested increased water intake to decrease pAVP levels, reporting better therapeutic results compared with the usual medications. To investigate easier strategies for maintaining low pAVP levels, we conducted a randomized controlled trial that assessed the outcomes of new therapeutic interventions, namely the management of vasopressin secretion for the treatment of Meniere's disease. In this previous study, interventions such as abundant water intake, tympanic ventilation tubes, and sleeping in the darkness could be feasible to decrease vasopressin secretion (20). These findings suggest that there may be alternative effective treatments for Meniere's disease to maintain low pAVP levels.

**Figure 5:**
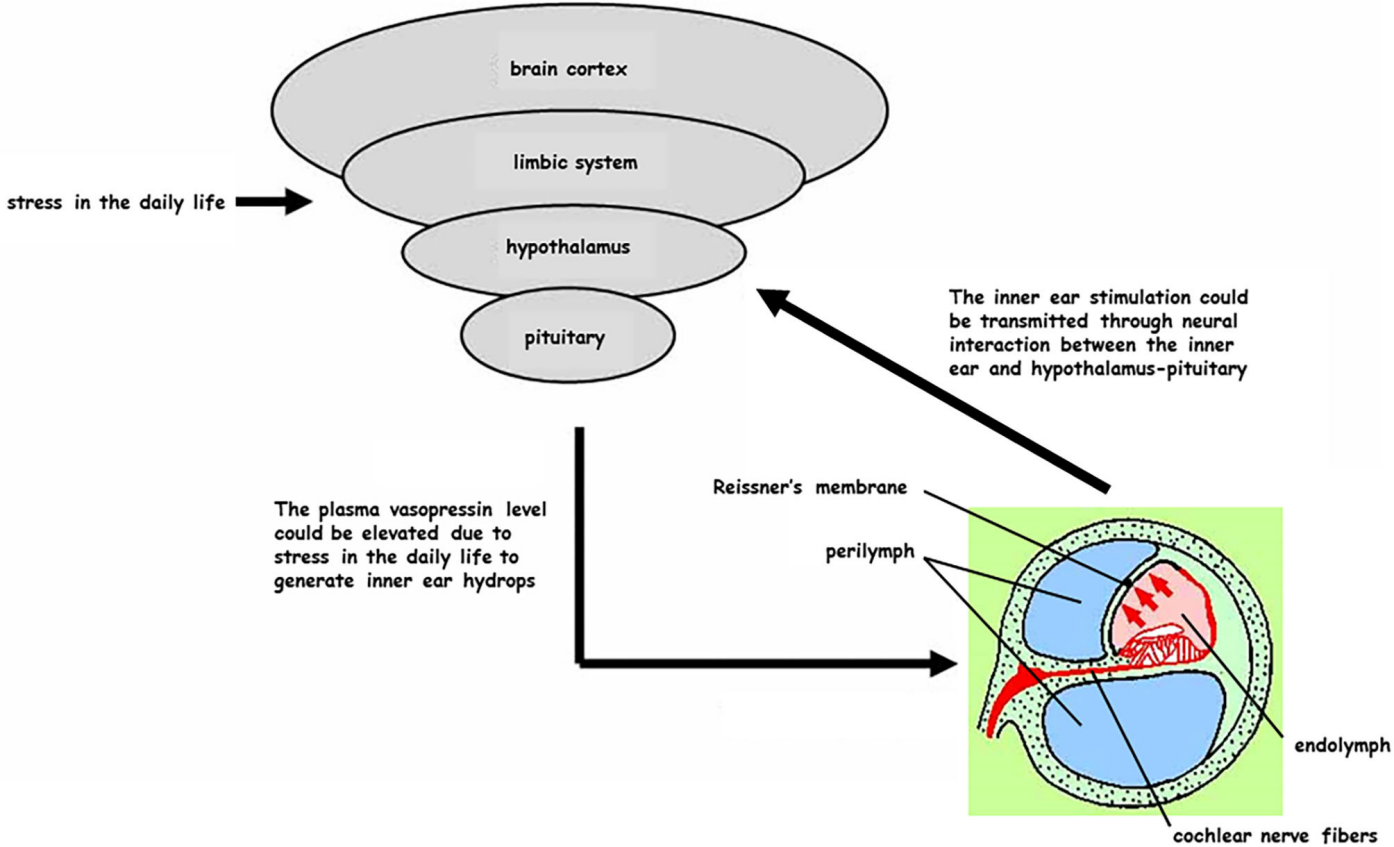
Reciprocal interaction between the inner ear and hypothalamus-pituitary system (partial hypothesis). Civilized people are frequently exposed to stress in their daily life and plasma vasopressin (pAVP) could become elevated at any time to generate the inner ear endolymphatic hydrops. On the other hand, some treatments for the inner ear could be transmitted as a stimulation through the neural interaction from the inner ear to hypothalamus-pituitary.

In today's society it is difficult to lead a stress-free life, particularly in terms of finding a non-stressful job, having enough time to relax, and getting enough quality sleep. In intractable Meniere's disease cases, we recommend surgical treatment as a secondary strategy. Since surgical procedures place physical and mental stress on patients, pAVP levels generally increase and urine volume decreases during surgery. On the contrary, surgical procedures involving the middle ear and inner ear may result in a reduction in pAVP levels and an increase in urine volume during surgery (21). In the present study, endolymphatic sac drainage surgery involved the following processes: drilling the mastoidectomy, exposing the posterior fossa dura, opening the endolymphatic sac, and placing a high concentration of steroids around the opened endolymphatic sac (15, 16). In our study, pAVP levels were reduced immediately after endolymphatic sac drainage surgery, and this reduction was much greater than that seen in patients with chronic otitis media after tympano-mastoidectomy. These findings suggest that procedures associated with endolymphatic sac drainage surgery, from posterior fossa dura exposure to intra-endolymphatic sac treatment, may result in a reduction of pAVP levels, although there is no evidence these procedures actually reduce pAVP levels. However, direct connections between the inner ear endorgans and the hypothalamus-pituitary system have been demonstrated, not only physiologically (22) but also morphologically (23). These findings suggest that inner ear stimulation could transmit to the hypothalamus-pituitary axis to modulate AVP production and/or secretion (Figure 5).

Since Yamakawa (2) and Hallpike and Cairns (3) discovered endolymphatic hydrops in Meniere's disease the development of inner ear gadolinium-enhanced MRI has been a useful advance in inner ear imaging (24, 25). However, neither oto-pathologic observation nor MRI findings can prove that endolymphatic hydrops causes Meniere's symptoms. The causal mechanisms of endolymphatic hydrops and the onset of Meniere's symptoms remain unclear, so symptomatic treatments might be preferable to medications and to surgery in particular.

In the present paper, we propose that pAVP-V2R activation in the inner ear could be one of the causes of endolymphatic hydrops and subsequent Meniere's attacks. In the modern era, it is difficult to avoid stress and maintain low levels of pAVP. Before considering molecular targeted therapy for AVP-V2R, we should implement simpler strategies for maintaining low levels of pAVP as outlined in basic studies of the molecular mechanisms of inner ear fluid homeostasis.

## Data Availability Statement

The original contributions presented in the study are included in the article/supplementary material, further inquiries can be directed to the corresponding author/s.

## Ethics Statement

The studies involving human participants were reviewed and approved by the Ethics Committee of Osaka University Hospital. The patients/participants provided their written informed consent to participate in this study. Written informed consent was obtained from the individual(s) for the publication of any potentially identifiable images or data included in this article.

## Author Contributions

TK and TO designed the research. TI, HF, and KU performed the research. TK analyzed the data and wrote the paper. All authors contributed to the article and approved the submitted version.

## Funding

This study was supported in part by JSPS KAKENHI Grant (2020–2022) from the Japan Agency for Medical Research and Development (AMED) under Grant Number 18dk0310092h000a and Health and Labor Sciences Research Grant for Research on Rare and Intractable Diseases [R02-Nanchito (Nan)-Ippan-04] from the Ministry of Health, Labor and Welfare of Japan.

## Conflict of Interest

The authors declare that the research was conducted in the absence of any commercial or financial relationships that could be construed as a potential conflict of interest.

## Publisher's Note

All claims expressed in this article are solely those of the authors and do not necessarily represent those of their affiliated organizations, or those of the publisher, the editors and the reviewers. Any product that may be evaluated in this article, or claim that may be made by its manufacturer, is not guaranteed or endorsed by the publisher.
